# Protein Disulfide-Isomerase Interacts with a Substrate Protein at All Stages along Its Folding Pathway

**DOI:** 10.1371/journal.pone.0082511

**Published:** 2014-01-20

**Authors:** Alistair G. Irvine, A. Katrine Wallis, Narinder Sanghera, Michelle L. Rowe, Lloyd W. Ruddock, Mark J. Howard, Richard A. Williamson, Claudia A. Blindauer, Robert B. Freedman

**Affiliations:** 1 School of Life Sciences, University of Warwick, Coventry, United Kingdom; 2 Department of Chemistry, University of Warwick, Coventry, United Kingdom; 3 School of Biosciences, University of Kent, Canterbury, United Kingdom; 4 Department of Biochemistry, University of Oulu, Oulu, Finland; Universitat Autònoma de Barcelona, Spain

## Abstract

In contrast to molecular chaperones that couple protein folding to ATP hydrolysis, protein disulfide-isomerase (PDI) catalyzes protein folding coupled to formation of disulfide bonds (oxidative folding). However, we do not know how PDI distinguishes folded, partly-folded and unfolded protein substrates. As a model intermediate in an oxidative folding pathway, we prepared a two-disulfide mutant of basic pancreatic trypsin inhibitor (BPTI) and showed by NMR that it is partly-folded and highly dynamic. NMR studies show that it binds to PDI at the same site that binds peptide ligands, with rapid binding and dissociation kinetics; surface plasmon resonance shows its interaction with PDI has a K_d_ of ca. 10^−5^ M. For comparison, we characterized the interactions of PDI with native BPTI and fully-unfolded BPTI. Interestingly, PDI does bind native BPTI, but binding is quantitatively weaker than with partly-folded and unfolded BPTI. Hence PDI recognizes and binds substrates via permanently or transiently unfolded regions. This is the first study of PDI's interaction with a partly-folded protein, and the first to analyze this folding catalyst's changing interactions with substrates along an oxidative folding pathway. We have identified key features that make PDI an effective catalyst of oxidative protein folding – differential affinity, rapid ligand exchange and conformational flexibility.

## Introduction

To understand how protein folding occurs in living cells we need to be able to describe protein folding pathways in the presence of cellular folding factors, and to characterize interactions between these factors and their unfolded and partly-folded protein substrates in thermodynamic, kinetic and structural terms. Such studies are not yet well-developed. In this paper we have selected unfolded, partly-folded and folded species corresponding to stages on a well-defined oxidative protein folding pathway and characterized in physico-chemical terms their interaction with PDI, the major catalyst of oxidative protein folding in the endoplasmic reticulum of eukaryotic cells.

Oxidative protein folding is a special case of protein folding in which the process of ‘conformational folding’ is accompanied by the formation of disulfide bonds (disulfide generation). The process occurs in several cellular compartments [1], but has been most extensively studied in relation to proteins secreted by eukaryotic cells, whose oxidative folding occurs primarily in the endoplasmic reticulum [2].

For over 50 years, studies *in vitro* on the refolding of purified proteins from the reduced, unfolded state have played a significant role in defining ideas about protein folding in general [3], [4]. For several small proteins, oxidative protein folding pathways have now been defined, in terms of the identity of disulfide-bonded intermediates and their conformations [5]–[9]. In every case, generation of native disulfides involves both disulfide formation and disulfide isomerization steps; rate-determining steps frequently involve the concomitant formation of ‘correct’ buried disulfide bonds and of the native conformation [3], [10].

Enzymatic catalysis of oxidative folding was first described in 1963 [11], [12] and since then the key catalyst, protein disulfide-isomerase (PDI), has been extensively characterized [13]. PDI catalyzes oxidative folding of many substrates including proteins with well-defined oxidative folding pathways such as ribonuclease [14], [15], lysozyme [16] and basic pancreatic trypsin inhibitor (BPTI) [17]–[19]. In each case, PDI catalyzes formation and isomerization of disulfides, both in early unstructured intermediates, and in later rate-determining steps in which relatively structured intermediates undergo disulfide isomerization to form stable conformation and stable native disulfides. These rate-determining steps require partial unfolding of structured intermediates, so their catalysis by PDI implies that it interacts with substrate transition states which are less folded than either ‘structured intermediate’ reactants or fully folded products (consistent with PDI having ‘chaperone’ activity independent of its ability to catalyze thiol∶disulfide interchanges [13]).

Studies on PDI catalysis of oxidative protein folding have focused on defining the enzyme-catalyzed disulfide-generation pathway of the substrate protein but have not analyzed interactions between the substrate and catalyst to provide understanding of how this interaction changes through the folding pathway. In this paper, we describe studies on the interaction between PDI and the small disulfide-bonded protein BPTI, using derivatives of BPTI selected to model stages on its oxidative folding pathway.

**Figure 1:**
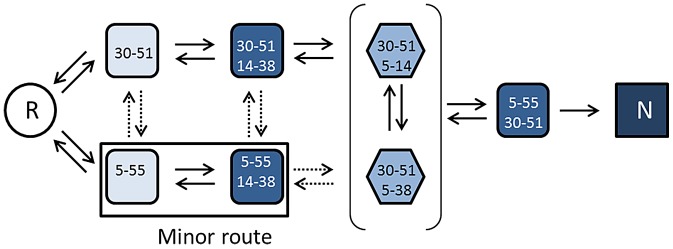
Disulfide generation pathway for oxidative folding of reduced BPTI. The figure represents species and steps on the pathway from reduced BPTI (R, circled) to generate native BPTI (N, square box). Intermediates are identified in terms of the disulfide bonds that are present with colour intensity reflecting the extent of native conformation. The intermediates in hexagonal boxes contain a non-native disulfide and occur transiently in the pathway between the predominant 2-disulfide intermediates and the final 2-disulfide intermediate. Other intermediates (in rounded boxes) contain only native disulfide bonds (either one or two). Solid arrows represent steps that can occur via a single thiol∶disulfide interchange reaction (isomerization or oxidoreduction with an exogenous thiol/disulfide reagent). Dashed arrows represent hypothetical steps in which one protein disulfide is lost and a different one is formed, requiring a minimum of two thiol∶disulfide interchanges.

Native BPTI contains two buried disulfides (linking residues 30–51 and 5–55) and one exposed disulfide (linking residues 14–38); its disulfide regeneration pathway has been the subject of considerable work and some contention (Figure 1). Pioneering work in the 1970s [20], [21] established that it was a restricted pathway with few defined intermediates; the 30–51 disulfide was predominant in species containing a single disulfide and the rate-determining step on the oxidative folding pathway was the formation of the two-disulfide intermediate containing both buried native disulfides (30–51+5–55) but lacking the exposed disulfide. Intriguingly, this species could not be formed directly from either of the one-disulfide intermediates containing a single native disulfide, but only via disulfide rearrangement of alternative two-disulfide intermediates. Later work [22] re-quantified the levels of the intermediates, leading to a revised pathway focusing on the quantitatively predominant two-disulfide intermediates, those containing two ‘native’ disulfide bonds ((30–51+14–38) and (5–55+14–38). However, the rate-determining steps remained disulfide isomerizations at the two-disulfide stage leading to formation of the (30–51+5–55) species. Species containing a non-native disulfide are crucial transient intermediates on the disulfide regeneration pathway, as originally proposed [23], [24].

The two-disulfide BPTI species containing the ‘native’ 30–51 disulfide and the ‘non-native’ 5–14 disulfide is one of these transient intermediates in the disulfide isomerization pathway; it can be formed by isomerization from the predominant two-disulfide intermediates and can isomerize in a single step to yield the final intermediate. A stable analogue of this species can be generated by mutation or modification of Cys38 and Cys55. Previous work on such a double mutant (C38S/C55S) indicated that it had a partly-folded conformation [6]. In this study, we have extended this earlier work and selected this protein as a model of a partly-folded intermediate on the BPTI oxidative folding pathway. We have studied its interaction with PDI and compared this interaction with those of native, folded BPTI and of reduced/alkylated BPTI (selected as a model of the fully unfolded protein). Hence we have compared the interactions of PDI with a series of proteins representing stages along an oxidative protein folding pathway.

## Methods

### Recombinant Constructs, Mutagenesis and Expression

Recombinant proteins were expressed in *E. coli* BL21 (DE3) pLysS strains from a vector derived from pET23b (Novagen). BPTI constructs were expressed with a leading methionine, used as a start codon, but the wild-type was otherwise identical to mature BPTI. The wild-type BPTI construct, and details of expression, isolation and solubilisation of inclusion bodies were described previously [25]. To generate Cys-to-Ser mutations, PCR was performed using PfuUltra High-Fidelity DNA Polymerase (Agilent) to amplify the entire vector, following manufacturers' guidelines. PDI constructs included a His-tag sequence (MHHHHHHM) to facilitate purification and were expressed and purified as described previously [26]. To produce ^15^N-labeled proteins, cells were grown in minimal medium containing 1 g/l of ^15^N ammonium sulfate.

### Preparation of BPTI and derivatives

Solubilized BPTI inclusion bodies were centrifuged, supernatant was collected and reduced BPTI was isolated by desalting through 4×5 ml HiTrap columns (GE Healthcare), attached to an ÄKTApurifier 100 FPLC, eluting with 10 mM HCl.

Both wild-type and mutant (C38S/C55S) BPTI were subjected to oxidative refolding, by dilution with 10 mM HCl and gradual addition (with stirring) of refolding buffer to a final protein concentration of 50 µM (0.3 mg/ml). The refolding buffer was 0.1 M Tris-HCl, 1 mM EDTA, pH 8.7 with 0.5 mM glutathione disulfide (GSSG) and 2 mM glutathione (GSH). The mix was incubated with gentle stirring overnight at 4°C before centrifugation at 75,000× g. To generate reduced/alkylated BPTI, reduced wild-type protein (50 µM) was incubated with 50 mM iodoacetamide in 100 mM sodium phosphate pH 7.0 at room temperature for 20 min.

All BPTI proteins were concentrated and reagents removed by solid phase extraction (SPE) using pre-packed 3 ml Grace Vydac SPE columns, containing reverse phase C-18 bonded to 13 µm silica beads with a 300 Å pore diameter. Protein samples were prepared following the manufacturer's instructions and applied to equilibrated columns by gravity flow overnight at 4°C. Acetonitrile was evaporated from the eluted protein in a vacuum desiccator overnight. The protein sample was then lyophilized for 4–6 hours and stored at −20°C.

Authentic BPTI (from bovine pancreas) was sourced from Sigma-Aldrich.

### Mass spectrometry

Masses of various forms of wild-type and mutant BPTI were determined by electrospray ionization mass spectrometry on either a Bruker microTOF or a Synapt G2 Q-Tof instrument. Oxidatively-refolded and reduced/alkylated species were generated as described above. Refolded/alkylated material was generated by reaction of the refolded protein (50 µM) with 5 mM iodoacetamide in 20 mM phosphate buffer, pH 7.3 containing 8 M urea, for 20 mins in the dark at room temperature before quenching with 20% acetonitrile/2% trifluoroacetic acid..

### Surface Plasmon Resonance

Surface plasmon resonance (SPR) was performed using a Biacore 2000 instrument (GE Healthcare). Proteins for immobilization were prepared at 50 µg/ml in HEPES-buffered saline containing EDTA and surfactant P20 (HBS-EP). A CM5 sensor chip was used, onto which proteins were immobilized by amine coupling, with pH scouting determining an optimal pH of 5.0. The standard protocols were followed using the manufacturer's instructions for both amine coupling and binding analysis. Analytes for binding analysis were prepared in HBS-EP running buffer to a volume of 370 µl for 2 replications. Analyte was injected at a flow rate of 50 µl/min. BIAevaluation version 3.1 was used to process and analyze the raw sensorgram data. Sensorgrams were baseline corrected using the Y-transform function. All curve fitting used simultaneous k_a_/k_d_ calculations and the 1∶1 (Langmuir) binding model was deemed the most appropriate model after parameter optimization. Analyses assuming a heterogeneous ligand provided two dissociation constants per ligand, but the K_D_ values generated were so close as to suggest that the simpler binding model with a single dissociation constant was more appropriate.

### NMR Spectroscopy

Unless otherwise stated, the NMR buffer used was 25 mM sodium phosphate, 100 mM NaCl, 10% D_2_O, pH 6.5. All BPTI experiments were performed on a Bruker AV II 700 MHz spectrometer, using a TCI cryoprobe with Z gradients (Bruker). For PDI **bb'x** samples, HSQC spectra were acquired at 298 K using a four channel Varian Unitylnova 600 MHz NMR spectrometer, equipped with a 5 mm HCN z-pulse field gradient probe. Both 2D TOCSY and 2D NOESY experiments were performed using 4096 data points in the F2 dimension and 512 data points in the F1 dimension, with mixing times of 60 ms (TOCSY) and 100 ms (NOESY). Unless otherwise stated, HSQC experiments were performed using 2048 data points in the F2 dimension (^1^H) and 128 data points in the F1 dimension (^15^N). For PDI titrations into BPTI, ^15^N-labelled BPTI was used with molar ratios ranging from 200∶1 BPTI∶PDI to 1∶1 BPTI∶PDI. For wild-type and mutant BPTI, HSQC spectra were obtained using 0.4 mM samples. Due to its limited solubility, HSQC spectra of reduced/alkylated BPTI were acquired using 0.1 mM samples. To compensate for its lesser solubility, the number of scans was increased 5-fold for reduced/alkylated BPTI. BPTI titrations into PDI **bb'x** were obtained using 0.25 mM **bb'x**. HSQC spectra using wild-type BPTI and mutant BPTI used **bb'x**∶BPTI ratios of 25∶1, 5∶1 and 1∶1. Due to the limited solubility of reduced/alkylated BPTI, HSQC spectra were obtained using 0.25 mM **bb'x** at **bb'x**∶BPTI ratios of 25∶1 and 8.3∶1.

For hydrogen/deuterium exchange, the NMR buffer used 100% D_2_O as the solvent (25 mM sodium phosphate, 100 mM NaCl, 100% D_2_O, pH 6.5). All experiments were performed at 278 K. To allow a greater frequency of collection of HSQC spectra within the first hour of the time-course, the acquisition time was decreased by halving the number of data points per scan in the F1 dimension.

Data analyses were carried out using CcpNMR Analysis version 2 [27]. NMR assignments from Biological Magnetic Resonance Data Bank (BMRB) entry 5359 [28] were used to help assign the wild-type BPTI spectrum. Likewise, a previous study of PDI **bb'x** [26] provided assignments for **bb'x** control spectrum (BMRB entry 15974). To assign the backbone resonances for the mutant BPTI, 3D ^15^N NOESY-HSQC and 3D ^15^N TOCSY-HSQC experiments were performed.

## Results

### A mutant BPTI mimics a partly-folded intermediate in the oxidative folding pathway

We expressed mutant (C38S/C55S) BPTI in *E.coli*, recovered it from inclusion bodies and refolded it ([25], see Methods). The product was homogeneous and the mass was 6613 Da (Table 1, Figure S1), consistent with the presence of two Cys→Ser mutations and the formation of two disulfide bonds between the remaining Cys residues. This was confirmed by the fact that there was no change in mass when the refolded mutant was subjected to alkylation in denaturing conditions, whereas alkylation of the reduced mutant led to incorporation of 4 carboxamidomethyl groups (Table 1). Hence the refolded mutant protein contained no buried –SH groups.

**Table 1:** Deconvoluted Average Masses (Da) of various BPTI species.

	Wild-type	C38S/C55S double mutant
Reduced	6649.3	6616.4
Reduced/alkylated	6991.5*	6844.9
Refolded	6643.0*	6613.1*
Refolded/alkylated	6641.9	6611.8

Masses were determined by electrospray ionization mass spectrometry. Data marked * are means of 3 independent determinations all falling within +/−1 Da; the remaining data are single determinations. Example deconvoluted spectra for the refolded species are shown in Figure S1.

The observed mass of the refolded wild-type corresponds to the theoretical mass of fully-oxidised wild-type BPTI containing 3 disulfide bonds (6643 Da), while that of the refolded double mutant corresponds to the mass expected for the species containing 2 disulfide bonds (6613 Da). The mass differences between the refolded and reduced species confirm the formation of 3 and 2 disulfide bonds respectively. The mass of a carboxamidomethyl group (-CH_2_-CO-NH_2_) is 58 Da, hence the masses observed for reduced/alkylated wild-type and mutant are as expected for alkylation of 6 and 4 Cys residues respectively.

Alkylations of the refolded species were carried out in presence of 8M urea. No alkylated species were detected and hence any protein molecules containing free –SH groups must represent <1–2% of the total refolded material.

**Figure 2:**
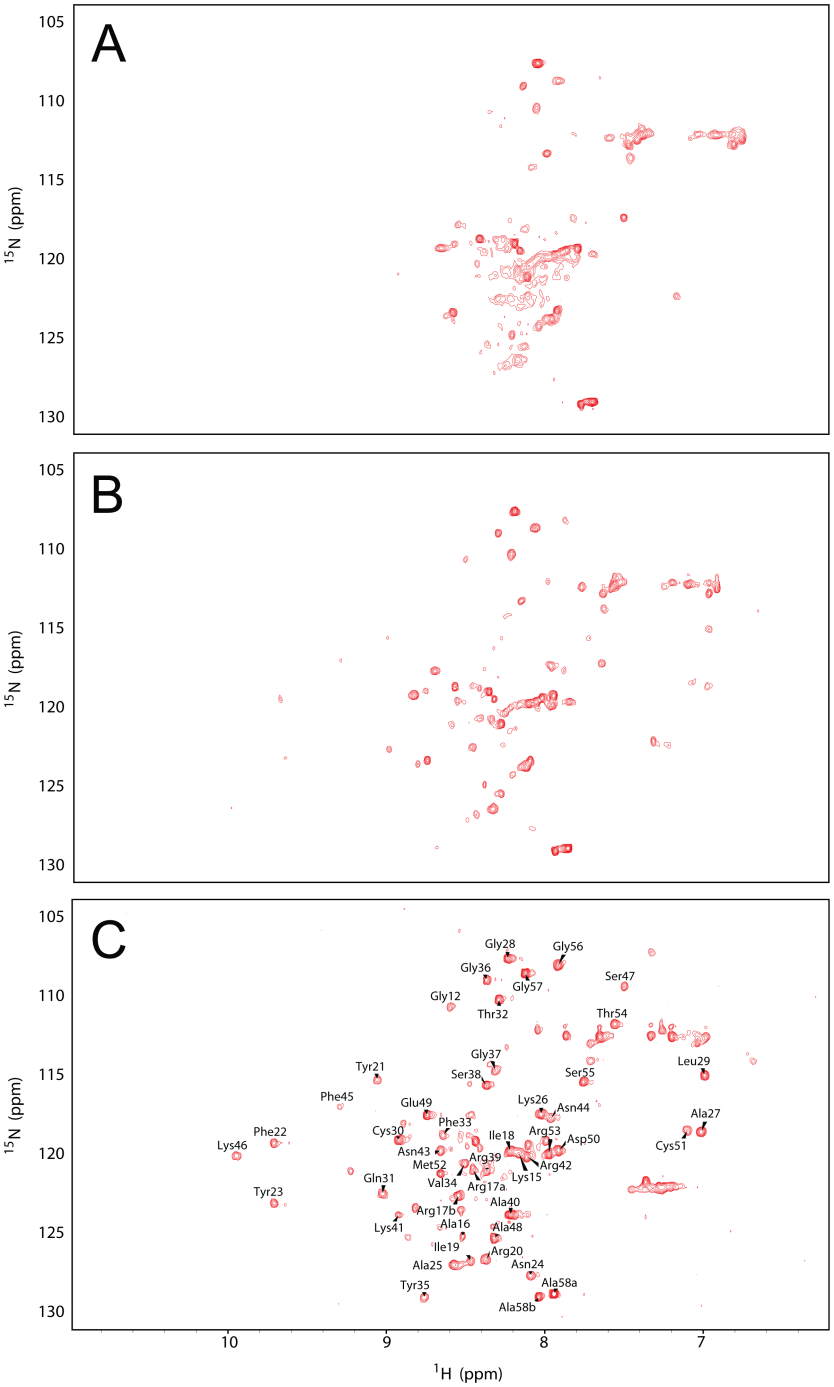
Temperature dependence of the ^1^H-^15^N HSQC spectrum of re-oxidized mutant BPTI. Spectra of re-oxidized C38S/C55S BPTI were collected at A) 36°C, B) 20°C and C) 5°C. Assignments are shown for resonances in the spectrum at 5°C.

For NMR studies, the protein was expressed in minimal medium and biosynthetically-labeled prior to oxidative refolding. ^1^H-^15^N HSQC spectra were collected at several temperatures. The HSQC spectrum at 36°C (Figure 2A) shows very poor dispersion and only a few well-resolved resonances, indicating little stable structure; at 20°C (Figure 2B) greater signal dispersion is evident and the spectrum at 5°C (Figure 2C) is well-dispersed with many well-resolved peaks, indicating the presence of a well-defined conformation. However, comparison between this HSQC spectrum and that of wild-type BPTI at the same temperature (Figure S2) shows extensive differences, so it was impossible to assign the mutant spectrum directly. To perform *de novo* assignment on the mutant, 2D ^1^H-^1^H TOCSY and ^1^H-^1^H NOESY spectra were collected at 5°C, and 3D ^15^N-edited NOESY/TOCSY experiments were performed to resolve ambiguities. This allowed assignment of all the well-resolved backbone amide resonances in the ^1^H-^15^N HSQC spectrum of mutant BPTI collected at 5°C (Figure 2C).

Interestingly, the amide resonances of residues 1–14 are not well-defined in this spectrum and could not be assigned (apart from Gly12), indicating that the N-terminal region is in intermediate exchange and not in a defined conformation even at 5°C, whereas the remainder of the molecule is well-folded. Hence the mutant has a partly-folded structure and will be referred to henceforward as ‘partly-folded BPTI’. The result is consistent with earlier work (at non-physiological pH 4.6), which showed that the NMR spectrum of this mutant was highly temperature-dependent and that it had a partly-folded conformation at −2°C which was lost at higher temperatures [6]. The NMR properties of the mutant are also very similar to that of a quadruple mutant (C5S/C14S/C38S/C55S) which contains only the native 30–51 disulfide bond [5], confirming that our mutant contains the native 30–51 plus the non-native 5–14 disulfide. Hence our ‘partly-folded BPTI’ corresponds to a transient intermediate on the oxidative folding pathway of the wild-type protein (cf. Figure 1).

### Comparison with native and unfolded BPTI

Recombinant wild-type BPTI was prepared similarly and the reduced protein was either re-oxidized or alkylated to block all six Cys residues (see Methods). Mass spectrometry (Table 1, Figure S1) and CD spectroscopy (not shown), and comparison with authentic BPTI from a commercial source, indicated the formation of fully-oxidized, folded BPTI and of alkylated, unfolded BPTI respectively.

No fully-oxidized form of BPTI containing non-native disulfides has ever been identified. However, to confirm beyond doubt that our oxidized refolded wild-type material was identical to native authentic BPTI, the 2D ^1^H-^1^H NMR spectra of reoxidized recombinant wild-type BPTI were recorded and compared with those of authentic non-recombinant BPTI. ^1^H-^1^H TOCSY and ^1^H-^1^H NOESY spectra were also used to derive resonance assignments. The assignments for the authentic and reoxidized recombinant proteins are shown overlaid in Figure S3. The close correspondence between the resonances recorded for authentic and reoxidized recombinant proteins (including for all Cys residues and their neighbours) provides confirmation – at the level of atomic resolution – that the recombinant material is fully oxidized and refolded.

**Figure 3:**
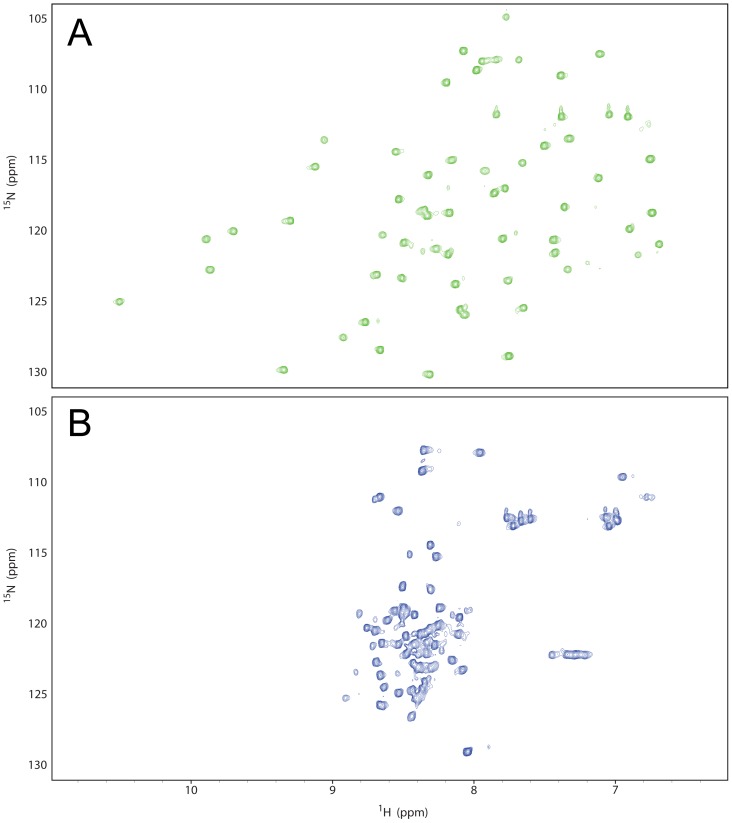
Comparison of the ^1^H- ^15^N HSQC spectra of folded and unfolded BPTI. Spectra of A) re-oxidized wild-type BPTI at 36°C, and B) reduced-alkylated wild-type BPTI at 5°C.

Recombinant wild-type BPTI was then biosynthetically ^15^N-labeled and prepared in both the oxidized and reduced/alkylated states for ^15^N-^1^H NMR analysis The ^15^N-^1^H HSQC spectrum of oxidized BPTI at 36°C (Figure 3A) shows sharp well-dispersed peaks characteristic of a native protein, confirming that the protein is folded under these conditions. The ^15^N-^1^H HSQC spectrum of reduced/alkylated BPTI at 5°C (Figure 3B), shows few, poorly-dispersed resonances with chemical shifts characteristic of random coil conformation; this material is essentially unfolded. Neither the oxidized/refolded nor the reduced/alkylated BPTI showed a significant temperature-dependence in their ^1^H-^15^N HSQC spectra over the range 5–36°C (data not shown), the former being folded and the latter unfolded throughout this range. These products will be referred to henceforward as ‘folded’ and ‘unfolded’ BPTI respectively. Figure S2 directly compares the ^15^N-^1^H HSQC spectra of folded, partly-folded and unfolded BPTI species at 5°C.

**Figure 4:**
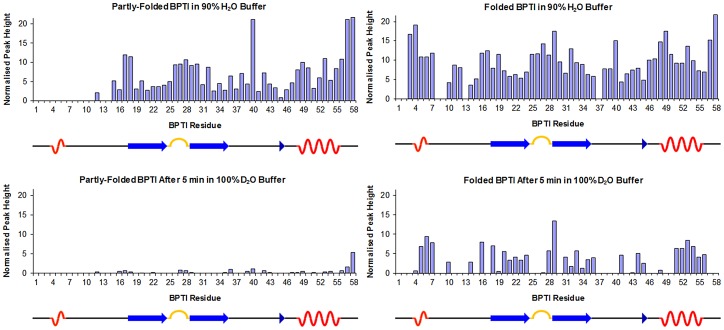
Comparison of H/D exchange properties of partly-folded and folded BPTI. Normalized peak heights (arbitrary units) for the assigned amide resonances of partly-folded BPTI (left panels) and folded BPTI (right panels) in 90% H_2_O buffer (upper panels) and after 5 mins in 100% D_2_O buffer (lower panels). Experiments were performed at 5°C. The peak heights are plotted against sequence number and a cartoon of the secondary structure of folded BPTI is shown below each panel.

The dynamics of the proteins were compared using H/D exchange at 5°C. For mutant partly-folded BPTI, almost complete exchange of backbone amide protons was observed after 5 minutes, in contrast to the wild-type folded protein where approximately 50% of backbone amide protons did not exchange rapidly (Figure 4). This indicates that the partly-folded conformation of the mutant is highly dynamic and lacks a stably-folded core. By contrast, the amides of 24 residues of the refolded wild-type BPTI are well-protected in these conditions and indicate the presence of a stable, folded core. The protected amides are found in regions that form secondary structure elements in the native protein (see cartoon of secondary structure beneath each panel in Figure 4) and the slowly-exchanging amides observed in this experiment at pH 6.5 correspond very closely to those that showed slow exchange in classic work conducted at pH 4.5 [29].

### Interaction of BPTI proteins with PDI

**Figure 5:**
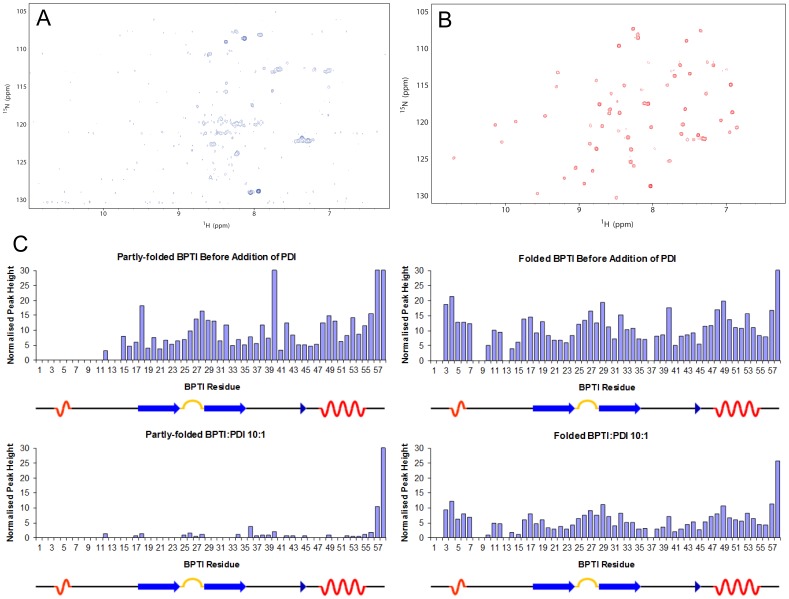
^1^H- ^15^N HSQC spectra of partly-folded and folded BPTI in presence of sub-stoichiometric PDI. The HSQC spectra, at 5°C in presence of 40 µM PDI, of A) 0.4 mM partly-folded BPTI and B) 0.4 mM native BPTI. C) The normalized peak heights (arbitrary units) for the assigned amide resonances of 0.4 mM partly-folded BPTI (left) and 0.4 mM native BPTI (right), plotted against the amino acid sequence number, in the absence of PDI (top panels) and in presence of 40 µM PDI (bottom panels). Below each panel is a cartoon representation of the secondary structure of native BPTI.

BPTI is small (<7 kD) compared to full-length PDI (>50 kD), so when unlabeled PDI is added to ^15^N-labeled BPTI species, their binding will result in a broadening and loss of intensity of the ligand resonances in heteronuclear NMR spectra. The interaction of ^15^N-labeled partly-folded BPTI (0.4 mM) with unlabeled PDI was studied at 5°C. In ^1^H-^15^N HSQC spectra, loss of ligand signal could be detected even at very low PDI concentrations (4 µM = 100-fold excess of BPTI, data not shown) and this effect became more pronounced as the PDI concentration was increased, so that no signal at all was detectable in presence of one molar equivalent of PDI (data not shown). Spectra of partly-folded BPTI in 50-fold excess (Figure S4A) and 10-fold excess (Figure 5A) confirm that loss of signal due to line broadening increases with PDI concentration. Furthermore, the significant effects observed even when the ligand is in considerable molar excess over PDI indicate that there is very rapid exchange between bound and free ligand. This result is unexpected in view of the size of the ligand.

To test if PDI is able to bind a fully-folded protein, labeled folded BPTI was used in a comparable experiment. There is very obvious loss of signal at the 1∶1 ligand∶PDI ratio (Figure S4B), but there is also some effect at the 10∶1 ratio (Figure 5B), which becomes clearer when signal intensities are quantified. Figure 5C shows quantitatively the signal intensities from partly-folded and folded BPTI when present in a 10-fold excess over unlabeled PDI compared to in absence of PDI. In both cases there is loss of signal on interaction with PDI but this is considerably more marked for the partly-folded BPTI.

Comparable experiments were performed by titrating PDI into the less soluble unfolded BPTI at 0.1 mM. Spectra at 50∶1 and 5∶1 excess of ligand over PDI (Figure S4C, S4D) may be compared with the spectrum in absence of PDI (Figure 3B); there is considerable loss of signal even at a 50-fold excess of ligand, consistent with a large fraction of unfolded BPTI binding to and dissociating from PDI on the timescale of the NMR experiment. The very significant loss of signal from all forms of BPTI when in presence of much lower concentrations of PDI implies rapid association and dissociation rates on a sub-millisecond timescale.

Clearly all forms of BPTI, even native BPTI, are recognized as ligands by PDI and the effects on signal intensity at different ligand∶PDI ratios show qualitatively that the affinity of PDI increases with increased protein ligand unfolding. To provide more quantitative data, PDI and a ligand-binding fragment of PDI (the **bb'x** domains [26]) were immobilized onto separate channels of a sensor chip for analysis of ligand-binding by surface plasmon resonance. The various forms of BPTI were applied at several temperatures; binding and dissociation curves were fitted simultaneously and analysed assuming the interactions can be described by 1∶1 binding stoichiometry with a single dissociation constant (see Methods). Table 2 presents the dissociation constants from these analyses which indicate clearly that affinity increases with increased ligand unfolding (i.e. K_D_ values increase from unfolded to partly-folded to native, folded BPTI) and that affinity of PDI for these ligands is greater at 36°C than at 5°C. Furthermore, affinities for these ligands are very similar for full-length PDI and for the **bb'x** fragment, reinforcing the conclusion that this fragment comprises the principal ligand binding site for peptides and unfolded proteins [30]–[32]. Reproducibility of the data is good, except for binding of folded BPTI at 5°C where the affinities are so low as to be difficult to determine with precision. Unfolded BPTI shows some non-specific affinity for an immobilized control protein (ovalbumin) but the affinities of partly-folded and folded BPTI for this control protein at 36°C are barely measurable, (K_D_ values 200–300 fold greater than for PDI) (data not shown).

**Table 2:** Dissociation constants (µM) for interaction of various BPTI species with PDI.

Temperature	5°C	5°C	36°C	36°C
Immobilized PDI	**Full-length**	**bb'x**	**Full-length**	**bb'x**
BPTI Ligand				
Unfolded	3.24, 3.29	3.17, 3.22	1.52, 1.87	1.88, 2.21
Partly-folded	13.6, 14.4	13.0, 16.0	4.07, 5.72	5.02, 6.37
Native	394, 1470	101, 420	11.6, 18.8	10.5, 11.1

The results shown are duplicate individual determinations obtained by surface plasmon resonance.

### The protein ligand interaction site on PDI

**Figure 6:**
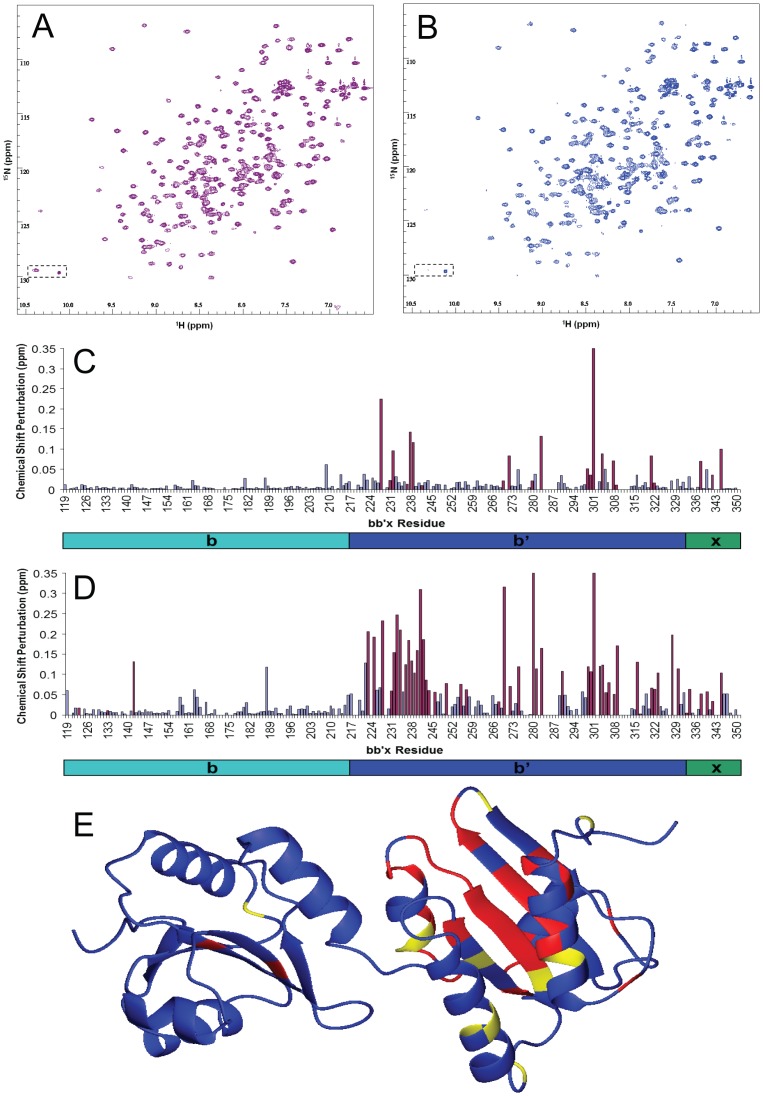
Identification of the site on PDI that interacts with partly-folded BPTI. ^1^H-^15^N HSQC spectra of 0.25 mM ^15^N-labeled **bb'x** fragment of PDI recorded at 25°C, A) in absence of any ligand, and B) in presence of 50 µM partly-folded BPTI. Peaks for the indole NH resonance of Trp 347 are highlighted within a dashed box. Chemical shift perturbations were measured from peaks in the spectrum in absence of ligand to the nearest peak found in the spectrum recorded in presence of ligand, and the net chemical shift perturbation was calculated as Δ^15^N/6+Δ^1^H. Net chemical shift perturbations were plotted versus sequence position in PDI, C) for data in presence of 50 µM ligand (panel B above) and D) for data in presence of 0.25 mM ligand (spectrum not shown). Blue bars represent shifts of peaks identified in the target spectrum, maroon bars represent shifts from peaks that could not be identified in the target spectrum, presumed to be due to line broadening. The cartoon below each graph indicates the position of each domain in the **bb'x** construct. The chemical shift perturbations seen in the presence of 0.25 mM ligand (panel D) are shown mapped onto the structure (PDB 2K18) in E), where red indicates perturbations >0.1 ppm and yellow indicates perturbations >0.06 ppm.

Previous work identified residues within the **b'** domain of PDI that form the binding site for peptides and unfolded proteins [26], [33]–[35], but no study has been done with a partly-folded intermediate on an oxidative protein folding pathway. ^15^N-labelled PDI **bb'x** was expressed and purified [26] and its interaction with unlabeled partly-folded BPTI was analyzed by collecting ^1^H-^15^N HSQC spectra of **bb'x** in absence and presence of mutant C38S/C55S BPTI (Figures 6A, 6B). Although many resonances are identical in the two spectra, others are either shifted or broadened. For example, the indole side-chain N-H proton resonance of W347 (boxed) shows a change in ^1^H shift from 10.4 ppm to 10.1 ppm. This change was seen previously on addition of a peptide ligand and results from displacement of the W347 side-chain from a hydrophobic site due to ligand binding [26], [33]. These spectra were analyzed by the ‘minimum-shift’ method [26], [36] (Figures 6C, 6D), showing that the effect of ligand-binding is focused on specific regions within the **b'** domain, with little effect on the **b** domain. Figure 6E represents the data structurally, showing that the binding site for partly-folded BPTI comprises mainly core β-sheet residues of the **b'** domain, corresponding to residues previously identified as the binding site for peptides and unfolded proteins [26], [34].

## Discussion

### PDI discriminates between substrates based on their degree of folding

The three proteins studied here showed contrasting conformational and dynamic properties. Reoxidized wild-type BPTI was folded at all temperatures and showed backbone amide H/D exchanges occurring over a range of timescales, with a core of well-protected amides. Reduced/alkylated BPTI behaved as an unfolded protein with an NMR spectrum showing poor dispersion at all temperatures. Oxidized mutant C38S/C55S BPTI, containing one native and one non-native disulfide, showed considerable temperature-dependence in its conformational properties, giving a well-defined assignable NMR spectrum only at 5°C or below. The N-terminal region of this species was not folded, even at low temperatures, and all backbone amides exchanged on a time-scale of 10^2^ seconds at pH 6.5; so this protein is only partly-folded and lacks a stably-folded core. Hence, the three proteins provide good models for species at different stages along an oxidative protein folding pathway.

NMR and SPR studies showed that all three proteins interact with PDI but with different affinities; the folded protein bound least tightly and the unfolded protein most tightly. Hence PDI's ability to discriminate between ‘substrates’ and ‘products’ arises from quantitative differences in its affinity for unfolded, partly-folded and fully-folded proteins. We infer that PDI selects and binds to unfolded regions in all substrates in a manner analogous to its binding of peptide ligands [26], [32], [33]. Each protein's conformation is dynamic and regions of folded protein transiently unfold and refold on timescales that reflect the stability of the folded state. Our data suggest that regions of unfolded polypeptide are selected preferentially as ligands by PDI and that the observed quantitative differences in affinity of PDI for the three substrates reflect their tendencies to local unfolding. It is possible that PDI recognizes the partly-folded ligand exclusively through its ‘unfolded’ region (residues 1–14), but the fact that native BPTI is also bound (albeit more weakly) suggests that transiently unfolded regions are recognized. This interpretation is supported by the fact that the affinities determined by SPR for all three proteins increase with temperature from 5°C to 36°C, consistent with the view that PDI binds unfolded and dynamic regions of its protein ligands, which become increasingly available as the temperature increases.

The SPR experiments also allowed a direct comparison of binding by full-length PDI and the **bb'x** fragment; there was no difference in the affinities observed, implying that any interactions made by these protein ligands with other regions of PDI – the **a**, **a'** and **c** domains – do not contribute significantly to the binding. In summary, we have shown that PDI makes similar reversible non-covalent interactions with a set of proteins that model an oxidative folding pathway; dissociation constants lie in the range between 10^−3^–10^−6^ M and dissociation rate constants are >10^3^ s^−1^.

### Interactions between PDI and ligands in the cell

How well does the system studied here represent PDI/substrate interactions which occur *in cellulo*? Complex disulfide isomerizations occur in the oxidative folding pathways of several proteins *in cellulo* [37], [38] but detailed studies of interactions with folding catalysts have not been attempted. We have studied these interactions in a model system and mimicked some of the conditions that hold *in vivo*. All NMR and SPR experiments were performed at approximately physiological pH and at temperatures from 36°C down to 5°C. Protein concentrations were in the 0.1–1 mM range (comparable to the concentration of PDI within the ER lumen) but pure proteins were used and no agents were added to mimic the overall crowding that occurs within the ER [39]. Crowding would lead to further folding of partly-folded species, so perhaps the partly-folded model protein used here would retain some folding at physiological temperature. Crowding would also increase the affinity of PDI for ligands in general [40], though it would not be expected to change the relative order of affinities.

A further issue relates to oxidants for protein disulfide formation in the ER. The non-catalyzed disulfide generation pathway of BPTI described above (Figure 1) was determined from experiments using small molecule disulfides as oxidants. The complexity of the disulfide isomerizations on the oxidative folding pathway of BPTI has been ascribed to the difficulty such disulfide oxidants have in accessing and oxidizing buried thiol groups. Recently, the oxidative folding pathway of BPTI was studied using H_2_O_2_ as oxidant and evidence was presented suggesting that this small highly reactive oxidant can effectively oxidize buried thiols, simplifying the disulfide generation pathway [41]. Furthermore, there is now a much clearer picture of the various routes by which oxidizing equivalents can flow via PDI to reduced proteins within the ER [42]. Enzymatic machinery exists within the ER to use both O_2_ and H_2_O_2_ as ultimate oxidants and to transfer oxidizing equivalents from them to PDI. Although various parts of this machinery have been reconstituted *in vitro* [43], [44], there has been no reinvestigation of disulfide regeneration pathways of protein substrates under these circumstances.

These issues bear on our observation that the same site on PDI is involved in binding peptides, unfolded proteins and partly-folded proteins. Can a substrate protein stay bound to PDI through the complete oxidative folding process, undergoing a series of disulfide formation and isomerization steps? Apparently this may depend on the oxidant. In the situation where the oxidizing equivalents are derived from O_2_ via the oxidase Ero1, substrate dissociation must occur between each oxidative step, since Ero1 binds to PDI via the ligand-binding **b'** domain [43] and may compete directly with other ligands for the same site [45]. Hence cycles of substrate and partner protein binding and release must occur in PDI turnover and these will be facilitated by the rapid dissociation rates that can be inferred from our observations. In other work [46], we are characterizing the flexibility and mobility of PDI and we observe extensive relative motion of its domains which may facilitate its ability to interact with a wide range of different substrate and partner proteins.

In conclusion, PDI is distinctive compared to thioredoxin or DsbA, with which it shares capabilities in thiol/disulfide redox chemistry [31], [47]. The key features enabling PDI to act as an effective catalyst of oxidative protein folding are i) its ability to bind to transiently- and permanently-unfolded regions of substrate proteins, through its promiscuous ligand binding site on the **b'** domain, ii) its capacity to distinguish folded, partly-folded and unfolded proteins through quantitative differences in binding affinity, iii) its extensive inter-domain flexibility allowing it to accommodate substrate and partner proteins of different shapes and sizes at the same promiscuous binding site, and iv) its ability to bind and release ligands on a sub-millisecond timescale, enabling it to change partners rapidly.

## Supporting Information

Figure S1 **ESI mass spectra of fully oxidised BPTI.** A) Wild type BPTI; B) BPTI containing C38S and C55S mutations.
(TIF)

Figure S2 **Comparison of the ^1^H-^15^N HSQC NMR spectra of folded, partly-folded and unfolded BPTI.** HSQC spectra for the various BPTI species at 5°C are overlaid. Blue, reduced-alkylated BPTI (unfolded); red, oxidized mutant (C38S/C55S) BPTI (partly-folded); green, oxidized wild-type BPTI (folded). Assignments are shown for resonances in the spectrum of folded BPTI.
(TIF)

Figure S3 **Comparison of assigned ^1^H resonances for authentic BPTI and oxidatively refolded wild-type recombinant BPTI.** ^1^H NMR assignments for authentic (solid lines, diamond symbols) and recombinant (dotted lines, square symbols) wild-type BPTI. Backbone HN assignments (red) fall at the top of the chart (average chemical shift = 8.1 ppm), backbone Hα (purple) in the middle (average chemical shift = 4.4 ppm) and side-chain Hβ (yellow) at the bottom (average chemical shift = 2.4 ppm). Where more than one chemical shift is available (e.g. Gly Hα) the most downfield chemical shift was plotted in all cases. Connecting lines are only shown between residues that are immediately sequential in the sequence.
(TIF)

Figure S4 **^1^H-^15^N HSQC spectra of various forms of BPTI in presence of PDI.** HSQC spectra of A) 0.4 mM partly-folded BPTI in presence of 8 µM PDI, B) 0.4 mM folded BPTI in presence of 0.4 mM PDI, C), 0.1 mM unfolded BPTI in presence of 2 µM PDI, and D) 0.1 mM unfolded BPTI in presence of 20 µM PDI. All spectra were acquired at 5°C.
(TIF)
