# Supplementary figures and images for: Protein Disulfide-Isomerase Interacts with a Substrate Protein at All Stages along Its Folding Pathway
Source: PLoS One. 2014 Jan 20;9(1):e82511. doi: 10.1371/journal.pone.0082511 (PMC3896340; doi:10.1371/journal.pone.0082511)

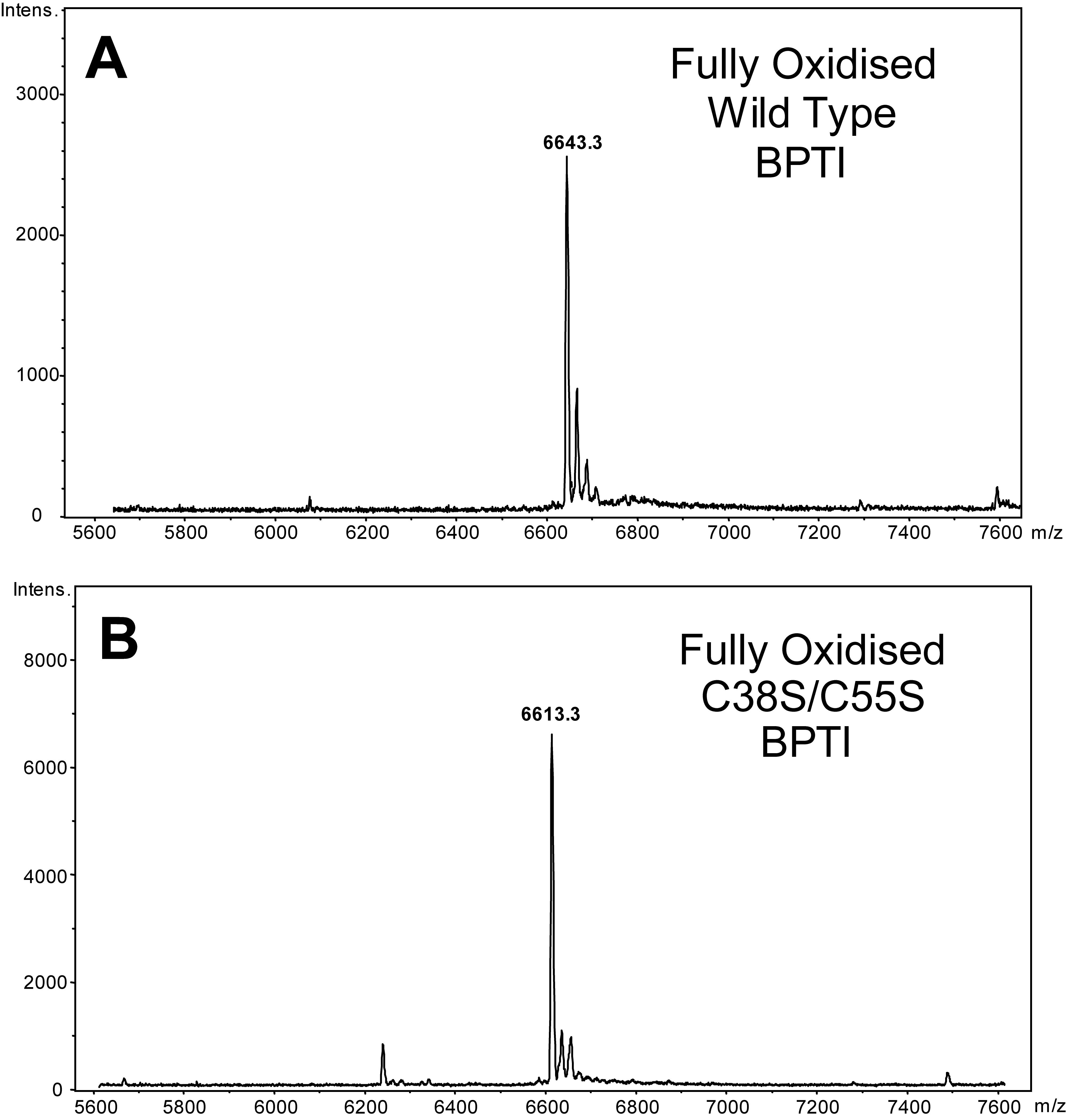

Supplement: Figure S1 — ESI mass spectra of fully oxidised BPTI. A) Wild type BPTI; B) BPTI containing C38S and C55S mutations. (TIF) [file pone.0082511.s001.tif]

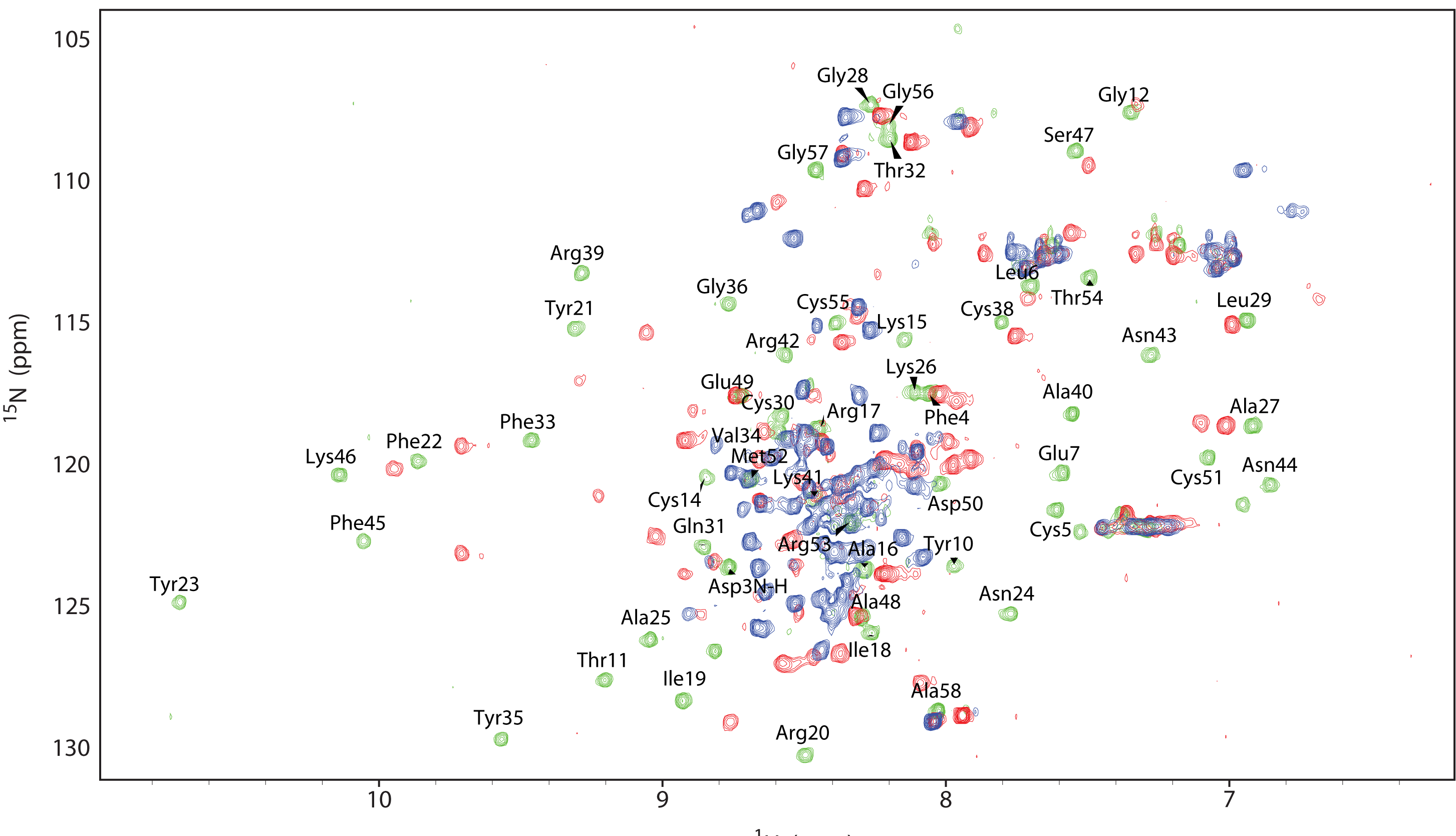

Supplement: Figure S2 — Comparison of the 1H-15N HSQC NMR spectra of folded, partly-folded and unfolded BPTI. HSQC spectra for the various BPTI species at 5°C are overlaid. Blue, reduced-alkylated BPTI (unfolded); red, oxidized mutant (C38S/C55S) BPTI (partly-folded); green, oxidized wild-type BPTI (folded). Assignments are shown for resonances in the spectrum of folded BPTI. (TIF) [file pone.0082511.s002.tif]

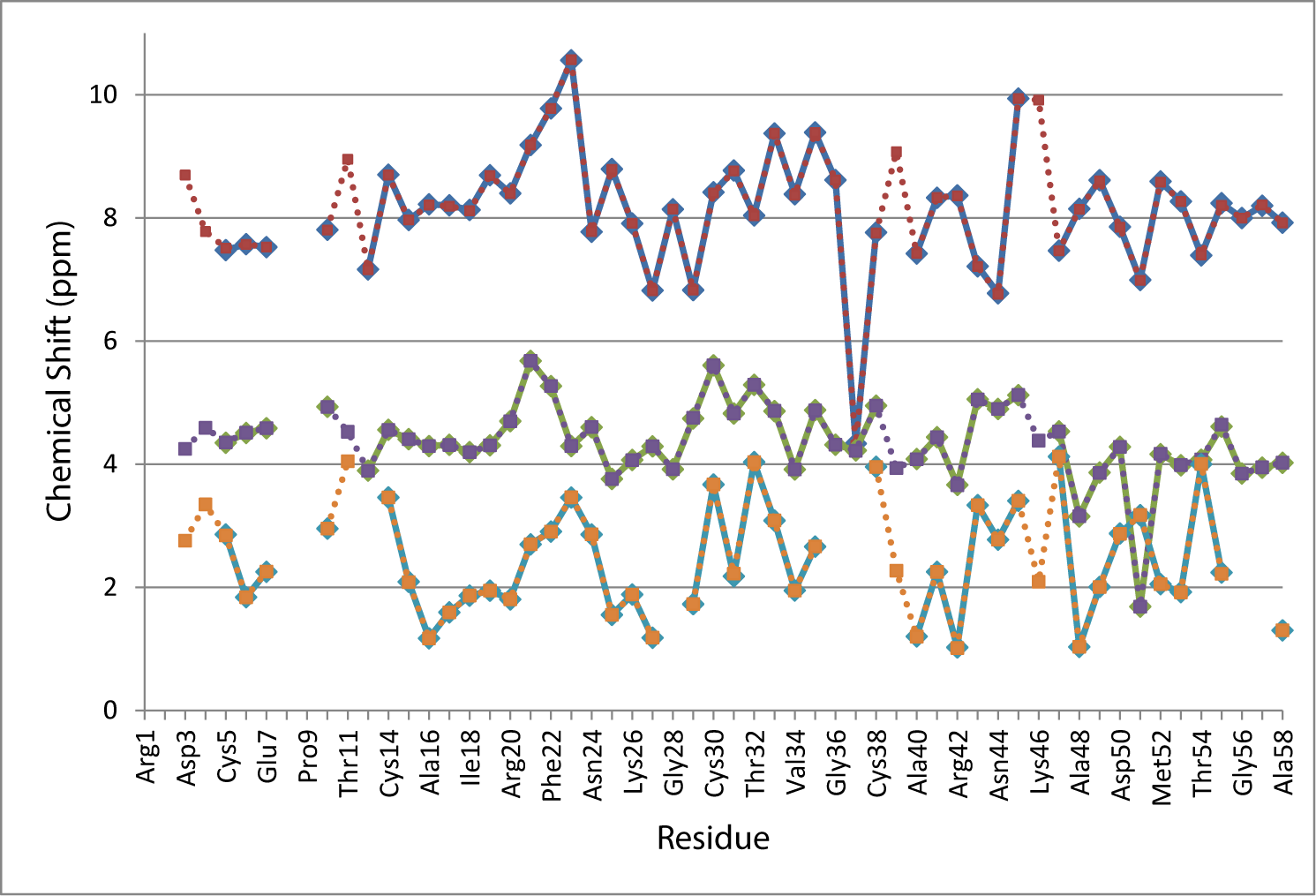

Supplement: Figure S3 — Comparison of assigned 1H resonances for authentic BPTI and oxidatively refolded wild-type recombinant BPTI. 1H NMR assignments for authentic (solid lines, diamond symbols) and recombinant (dotted lines, square symbols) wild-type BPTI. Backbone HN assignments (red) fall at the top of the chart (average chemical shift = 8.1 ppm), backbone Hα (purple) in the middle (average chemical shift = 4.4 ppm) and side-chain Hβ (yellow) at the bottom (average chemical shift = 2.4 ppm). Where more than one chemical shift is available (e.g. Gly Hα) the most downfield chemical shift was plotted in all cases. Connecting lines are only shown between residues that are immediately sequential in the sequence. (TIF) [file pone.0082511.s003.tif]

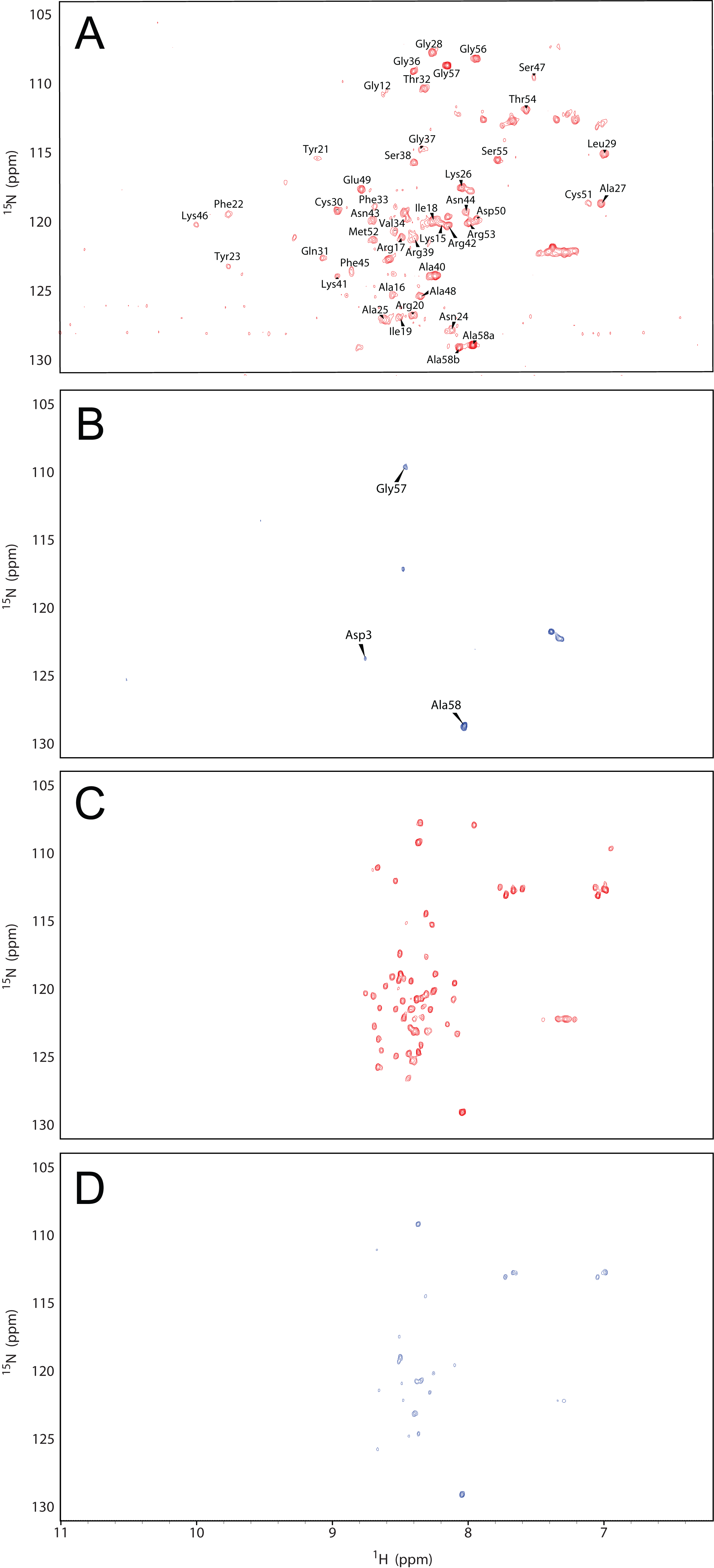

Supplement: Figure S4 — 1H-15N HSQC spectra of various forms of BPTI in presence of PDI. HSQC spectra of A) 0.4 mM partly-folded BPTI in presence of 8 µM PDI, B) 0.4 mM folded BPTI in presence of 0.4 mM PDI, C), 0.1 mM unfolded BPTI in presence of 2 µM PDI, and D) 0.1 mM unfolded BPTI in presence of 20 µM PDI. All spectra were acquired at 5°C. (TIF) [file pone.0082511.s004.tif]
